# Short version of the Problem Areas in Diabetes scale(PAID-13) in Brazilian patients with diabetes: a structural and criterion validity study

**DOI:** 10.1590/1516-3180.2024.0109.R1.03072024

**Published:** 2025-03-14

**Authors:** Daniel Santos Rocha, Almir Vieira Dibai-Filho, Abraão Albino Mendes, Nataly Borges da Costa Pinto, Kaiser Salgado Neves, Carlos Eduardo Neves Amorim, Plínio da Cunha Leal, Janaina de Oliveira Brito-Monzani, Daniela Bassi-Dibai

**Affiliations:** I Postgraduate Program in Physical Education, Universidade Federal do Maranhão (UFMA), São Luís, Brazil.; II Postgraduate Program in Physical Education, Universidade Federal do Maranhão (UFMA), São Luís (MA), Brazil.; III Postgraduate Program in Health Management and Care, Universidade Ceuma (UNICEUMA), São Luís (MA), Brazil.; IV Centro Universitário Santa Terezinha (CEST), São Luís (MA), Brazil.; V Centro Universitário Santa Terezinha (CEST), São Luís (MA), Brazil.; VI Postgraduate Program in Physical Education, Universidade Federal do Maranhão (UFMA), Pinheiro (MA), Brazil.; VII Postgraduate Program in Physical Education, Universidade Federal do Maranhão (UFMA), São Luís (MA), Brazil.; VIII Postgraduate Program in Physical Education, Universidade Federal do Maranhão (UFMA), São Luís (MA), Brazil.; IX Postgraduate Program in Health Management and Care, Universidade Ceuma (UNICEUMA), São Luís (MA), Brazil.

**Keywords:** Diabetes, Emotional distress, Surveys and questionnaires, Psychometric properties, Factor analysis, Clinimetrics

## Abstract

**BACKGROUND:**

The Problem Areas in Diabetes scale (PAID) is used to measure emotional distress levels related to diabetes mellitus (DM). However, consensus on its internal structure is lacking.

**OBJECTIVES:**

To compare the different internal structures of the PAID and propose a shortened version for Brazilian patients with diabetes.

**DESIGN AND SETTING:**

Structural and criterion validity study.

**METHODS:**

We included Brazilian patients with type 1 DM (DM1) and type 2 DM (DM2) in this study. In accordance with the international consensus recommendations, we assessed the structural validity using confirmatory factor analysis (CFA) and used the following indices to evaluate model fit: root mean square error of approximation (RMSEA), comparative fit index (CFI), Tucker–Lewis index (TLI), standardized root mean square residual (SRMR), chi-square/degrees of freedom (DF), Akaike information criterion (AIC) and sample-size adjusted Bayesian information criterion (SABIC). Modification indices and factor loadings were used to reduce the number of items.

**RESULTS:**

One hundred eighty-five patients, most of whom included women with DM2, participated in the study. The reduction in the PAID generated a unidimensional structure with 13 items (PAID-13). The PAID-13 presented the best-fit indices (chi-square/DF = 2.15, CFI = 0.989, TLI = 0.986, RMSEA = 0.079, and SRMR = 0.049). When the PAID versions with 13 and 20 items (original version) were correlated, a strong correlation was observed (rho = 0.941, P < 0.001).

**CONCLUSION:**

The short version of the PAID scale with 13 items presented a more appropriate internal structure for Brazilian patients with diabetes.

## INTRODUCTION

Patients with type 1 diabetes mellitus (DM1) and type 2 diabetes mellitus (DM2) frequently report emotional distress. The Problem Areas in Diabetes scale (PAID) is a 20-item self-assessment tool designed to measure emotional distress levels associated with diabetes mellitus (DM). The scale quantifies the perception and emotional experiences of patients with DM throughout their course of living with the disease. This instrument was created in 1995 in the United States.^1^

The PAID was translated into Portuguese language and validated for use in Brazil in 2007.^2^The authors found a high Cronbach’s alpha value, indicating satisfactory internal consistency with the original version. Furthermore, they evaluated the factorial structure using exploratory factor analysis (EFA), finding a single dimension; however, they chose to consider the PAID with four dimensions, as established by previous studies;^3^ therefore, there are no studies in the Brazilian literature that compare the different internal structures of the PAID.

In specialized literature, consensus regarding the dimensionality of this instrument is lacking. Several studies that validated the instrument for their respective countries evaluated the factorial structure by means of EFA and principal components analysis (PCA) and structures with four,^3^ three,^4^ two,^5,6^ and one domain^7^were found, in addition to a short version with 5 items,^8^ consensus regarding the best factorial structure for the PAID is lacking.

Lee et al.^9^ attempted to justify this disagreement: they stated that cultural differences could explain these findings. However, this hypothesis does not justify similar structures in studies from different countries and different dimensions in the same culture. Furthermore, most of these studies analyzed a single sample group, that is, individuals with DM1 and/or DM2, which may have influenced the results.^10^

Structural validity is an important psychometric property that measures whether results reflect the hypothetical dimensionality of a construct.^11^ EFA is used to identify correlations between defined variables and yet undefined factors, whereas confirmatory factor analysis (CFA) allows checking the correlation between variables and their respective factors in already validated or originally pre-established models.^12^

Given the instrument’s ability to measure emotional distress levels related to DM, we emphasize its relevance in scientific research and clinical applicability. However, no Brazilian study has compared the different factorial structures of the PAID to identify the most suitable one. In this study, we hypothesized that the short version of the PAID would present the best factorial structure, correlating strongly with the long version.

## OBJECTIVE

Considering the divergence between the dimensionality of the PAID and the lack of studies that verify the best Brazilian version for this scale, the objective of this study was to compare the different internal structures of the PAID using CFA and propose a short version for Brazilian patients with DM.

## METHODS

### Study design

This was a structural and criterion validation study. Data were collected online using Google Forms (Mountain View, United States). This research was disseminated through Facebook, Instagram, and WhatsApp (Meta Platforms Inc., Menlo Park, United States). All the participants provided virtual consent for inclusion in the study.

### Ethics approval and consent to participate

This study was approved by the Research Ethics Committee of the Universidade Ceuma, in São Luís (Maranhão, northeast Brazil) on August 29, 2018 (number 2.853.570) in accordance with the Declaration of Helsinki. All respondents freely participated in the study and signed an informed consent form.

### Participants and sampling

The sample size followed the recommendations of the COnsensus-based Standards for the selection of health Measurement INstruments (COSMIN): seven times the number of items in the questionnaire),^12^ which recommended a minimum of 140 individuals. The inclusion criteria were: men and women aged over 18 years, speakers of Brazilian Portuguese, and diagnosed with DM1 or DM2. Individuals diagnosed with cognitive changes and/or with the inability to read or write were excluded.

### Problems Areas in Diabetes scale (PAID)

The PAID is a self-administered self-report instrument originally developed at Joslin Diabetes Center in Boston, Massachusetts, USA. This scale assesses, from the patients’ perspective, the impact of diabetes and its treatment on their lives. The Brazilian version of the PAID comprises 20 items covering a range of emotional states frequently reported by patients with DM1 and DM2. The PAID produces a total score ranging from 0 to 100, with a high score indicating a high level of emotional distress. It uses a 5-point Likert scale ranging from: “Not a problem” = 0, “Small problem” = 1, “Moderate problem” = 2, “Almost a serious problem” = 3, “Serious problem” = 4. A total score of 0-100 was reached by summing the 0-4 responses given on the 20 items of the PAID and multiplying this sum by 1.25.^2^

### Statistical analysis

Descriptive statistical analysis was performed; the values are presented as means and standard deviations for quantitative variables and absolute numbers and percentages for qualitative variables. Descriptive analysis was performed using SPSS software version 17.0 (IBM, Chicago, United States).

We used CFA with the R Studio software (Boston, MA, USA) and the lavaan and semPlot packages. CFA was performed using a polychoric matrix and a robust diagonally weighted least squares (RDWLS) extraction method, as recommended by specialized literature for ordinal data.^13,14^ Model fit was assessed using the following indices: root mean square error of approximation (RMSEA) with 90% confidence interval (CI), comparative fit index (CFI), Tucker-Lewis index (TLI), standardized root mean square residual (SRMR), and chi-square/degrees of freedom (DF).^15,16^

In this study, values >0.90 were considered adequate for CFI and TLI, and values < 0.08 were considered adequate for RMSEA and SRMR. Values < 3.00 were considered adequate in the interpretation of chi-square/DF.^17,18^ To compare the PAID structures, the Akaike information criterion (AIC) and sample-size-adjusted Bayesian information criterion (SABIC) indices were used. The structure with the lowest AIC and BIC values was considered as the most parsimonious model, as recommended in the literature. In CFA, factor loadings ≥ 0.40 were considered adequate for the domain.^15,19^

We used modification indices and factor loadings to reduce the PAID. We considered items redundant in the PAID when the modification indices were greater than 10.0.^19,20^ In each pairwise analysis, we deleted redundant items with the lowest factor loadings.

Criterion validity was assessed by applying Spearman’s correlation coefficient (rho) between the PAID version with 13 items and the original version with 20 items (considered the gold standard according to COSMIN). Thus, criterion validity was met when rho ≥ 0.70.^12^

## RESULTS

We included 185 patients diagnosed with DM1 or DM2. The majority of the sample comprised women (75.1%) with a mean age of 49 years, married, with DM2 and with incomplete primary education. **Table 1** presents the personal and clinical characteristics of the patients.

**Table 1:** Patient characteristics (n = 185)

Variables	Mean (standard deviation) or number (%)
**Age (years)**	49.20 (14.90)
**Sex (female)**	139 (75.1%)
**Body mass (kg)**	71.53 (14.0)
**Stature (m)**	1.63 (0.07)
**Body mass index (kg/m** ^2^ **)**	26.91 (5.00)
**Diabetes**	
Type 1	54 (29.1%)
Type 2	131 (70.8%)
**Marital status**	
Single	54 (29.1%)
Married	117 (63.2%)
Divorced	7 (3.7%)
Widower	7 (3.7%)
**Schooling**	
Primary	81 (43.7%)
Secondary	37 (20.0%)
Superior	39 (21.8%)
Postgraduate	28 (15.1%)
**PAID**	
20 items (score, 0-100)	40.76 (24.95)
13 items (score, 0-100)	36.15 (28.07)

PAID = Problem Areas in Diabetes scale.

The original version of the PAID scale, with one domain and 20 items, was tested using CFA and presented inadequate fit indices, as shown in **Table 2**. Therefore, PAID reduction was performed based on the modification indices shown in **Table 3**, generating a unidimensional structure of the PAID scale with 13 items (PAID-13).

**Table 2:** Comparison between the different structures of the Problem Areas in Diabetes scale (PAID)

Structures	Chi-square/DF	CFI	TLI	RMSEA (90% CI)	SRMR	AIC	SABIC
Structure 1	2.15 *	0.989 *	0.986 *	0.079 (0.061, 0.098) *	0.049 *	6824.401	6825.993
Structure 2	4.88	0.913 *	0.902 *	0.145 (0.136, 0.155)	0.111	10844.783	10846.905
Structure 3	4.83	0.914 *	0.904 *	0.144 (0.135,0.154)	0.109	10824.593	10826.768
Structure 4	4.59	0.915 *	0.905 *	0.144 (0.134,0.154)	0.110	10810.865	10813.040
Structure 5	4.22	0.929 *	0.919 *	0.132 (0.122, 0.143)	0.103	10729.012	10731.293
Structure 6	4.22	0.931 *	0.919 *	0.132 (0.122, 0.143)	0.100	10686.191	10688.684
Structure 7	26.16	0.894	0.787	0.370 (0.317, 0.426)	0.193	2919.856	2920.387
**Factor loadings of the structure 1**							
Item 2 = 0.76							
Item 3 = 0.79							
Item 5 = 0.57							
Item 6 = 0.90							
Item 7 = 0.87							
Item 8 = 0.88							
Item 9 = 0.64							
Item 10 = 0.89							
Item 13 = 0.84							
Item 14 = 0.76							
Item 15 = 0.65							
Item 16 = 0.88							
Item 17 = 0.86							

DF = degree of freedom; CFI = comparative fit index; TLI = Tucker–Lewis index; RMSEA = root mean square error of approximation; CI = confidence interval; SRMR = Standardized Root Mean Squared Residual; AIC = Akaike information criterion; SABIC = Sample-size adjusted Bayesian information criterion.

Structure 1 = 1 domain and 13 items (items 2, 3, 5, 6, 7, 8, 9, 10, 13, 14, 15, 16 and 17);

Structure 2 = 1 domain and 20 items;

Structure 3 = 2 domains and 20 items (domain 1: items 1, 2, 14, 15, 16, 17, and 18; domain 2: items 3, 4, 5, 6, 7, 8, 9, 10, 11, 12, 13, 19 and 20);

Structure 4 = 2 domains and 20 items (domain 1: items 1, 2, 3, 4, 5, 6, 7, 8, 9, 10, 11, 12, 13, 14 and 19; domain 2: items 15, 16, 17, 18 and 20;

Structure 5 = 3 domains and 20 items (domain 1: items 1, 2, 3, 6, 7, 8, 9, 10, 12, 13, 14, 15, 16, 19 and 20; domain 2: items 4, 5 and 11; domain 3: items 17 and 18);

Structure 6 = 4 domains and 20 items (domain 1: items 3, 6, 7, 8, 9, 10, 12, 13, 14, 16, 19 and 20; domain 2: items 1, 2 and 15; domain 3: items 4, 5 and 11; domain 4: items 17 and 18);

Structure 7 = 1 domains and 5 items (items 3, 6, 12, 16 and 19).

* Adequate fit indices (chi/square/DF < 3, CFI and TLI > 0.90, RMSEA and SRMR < 0.08). Lower AIC and SABIC values indicate the best factor structure.

**Table 3:** Exclusion of redundant items from the Problem Areas in Diabetes scale (PAID)

Redundant Items	Item Description	MI	Factor Loading	Item Deleted
**Decision 1**				
Item 12	Worrying about the future and the possibility of serious complications?	198.023	0.632	Item19
Item 19	Coping with complications of diabetes?		0.508	
**Decision 2**				
Item 4	Uncomfortable social situations related to your diabetes care (e.g., people telling you what to eat)?	84.266	0.584	Item 4
Item 5	Feelings of deprivation regarding food and meals?		0.686	
**Decision 3**				
Item 5	Feelings of deprivation regarding food and meals?	41.272	0.686	Item 11
Item 11	Feeling constantly concerned about food and eating?		0.621
**Decision 4**				
Item 1	Not having clear and concrete goals for your diabetes care?	29.647	0.715	Item 1
Item 2	Feeling discouraged with your diabetes treatment plan?		0.795	
**Decision 5**				
Item 16	Feeling that diabetes is taking up too much of your mental and physical energy every day?	15.700	0.886	Item 20
Item 20	Feeling “burned out” by the constant effort needed to manage diabetes?		0.753	
**Decision 6**				
Item 17	Feeling alone with your diabetes?	14.215	0.866	Item 18
Item 18	Feeling that your friends and family are not supportive of your diabetes management efforts?		0.717	
**Decision 8**				
Item 7	Not knowing if your mood or feelings are related to your diabetes?	10.198	0.838	Item 12
Item 12	Worrying about the future and the possibility of serious complications?		0.632	

MI = Modification indices.

In this new scenario, the internal structure of PAID-13 (Structure 1) was compared with other structural models found in the literature, in addition to the original version (Structure 2). As described in **Table 2**, we also compared the following structures: structure with two domains and 20 items (structure 3) validated by Miller et al.;^6^ structure with two domains and 20 items (structure 4) validated by Veld et al.;^5^ structure with three domains and 20 items (structure 5) validated by Papathanasiou et al.;^4^ structure with four domains and 20 items (structure 6) validated by Snoek et al.;^3^ and structure with one domain and five items (structure 7) proposed by McGuire et al.^8^

Using CFA, adequate fit indices were found only in structure 1 of the PAID with 13 items (after adding four covariances to the model): chi-square/DF = 2.15, CFI = 0.989, TLI = 0.986, RMSEA = 0.079, and SRMR = 0.049. **Table 2** also presents the appropriate factor loadings (≥ 0.40) of the PAID-13 (structure 1). Regarding criterion validity, we observed a high correlation magnitude of 0.941 (P < 0.001) between the PAID versions with 13 and 20 items, even after reducing the number of items.

The Brazilian Portuguese version of the PAID-13 is available at https://questionariosbrasil.blogspot.com. To calculate the total PAID-13 score, all marked values were added, divided by 13, and multiplied by 25, generating a score ranging from 0 to 100 (higher scores indicating greater emotional distress).

## DISCUSSION

This study identified the most appropriate structure for the Brazilian version of the PAID with one domain and 13 items (PAID-13) using modification indices as a technique for reducing the instrument. This reduction was proposed after the original version of the PAID scale with one domain and 20 items presented inadequate fit indices.

The structure of the Brazilian version of the PAID was evaluated using EFA and only one domain was found.^2^ However, according to the authors, due to the small sample size, they opted to maintain a structure with 4 domains and 20 items—as established in the study conducted by Snoek et al.^3^ that investigated the dimensionality of the PAID in Dutch and North American patients with diabetes. These authors observed a suitable CFI for structures with one domain (0.93) and four domains (0.95).^3^ In our study, the CFI and TLI values were also adequate (> 0.90) for the same structures tested by Snoek et al.^3^ However, we reject both instrument structures, as we observed inappropriate values of RMSEA and SRMR. However, Snoek et al.^3^ did not investigate model residuals, which limits their analysis of the internal structure of PAID.

The PAID structure has been verified in several studies in different cultures, with different dimensions found in countries with the same culture and similar structures in different countries.^9^ Therefore, no consensus has been reached on the dimensions of this instrument. Our study is pioneering in proposing a new structure (the PAID-13) and comparing this new structure with existing structures using the AIC and SABIC fit indices.

Huang et al.^21^and Lee et al.^9^ pointed out some problems with the EFA used in most of these validation studies and recommended CFA as the best statistical evaluation method. Although the choice of EFA in these studies is justified by the fact that the original study did not evaluate this measurement property, there is a mistake by some authors when considering PCA as a synonym for EFA, which is not true. Our study used CFA to verify the dimensionality of the PAID using a methodology suitable for the instrument’s categorical ordinal responses (i.e., the polychoric correlation matrix and RDWLS extraction method).

This study has some limitations that must be considered. The sample primarily comprised women diagnosed with DM2. The proposal to reduce the PAID presented in this study was developed based on the Brazilian version of the instrument. Therefore, we recommend that PAID-13 be tested and validated in other languages. Notably, the PAID-13 score was derived from responses to the Brazilian version of the PAID with 20 items, which may have contributed to the high correlation between the PAID versions. In this sense, it is advisable that future research independently evaluate the criterion validity of the PAID-13, in addition to investigating its reliability, construct validity, and responsiveness.

## CONCLUSION

The short version of the PAID, with 13 items, presented a more adequate internal structure and excellent correlation with the long version. Therefore, this tool is recommended for use with patients with diabetes.
